# Supplementary material for: Integrated single-dose kinome profiling data is predictive of cancer cell line sensitivity to kinase inhibitors
Source: PeerJ. 2023 Nov 16;11:e16342. doi: 10.7717/peerj.16342 (PMC10657565; doi:10.7717/peerj.16342)
Supplement: Figure S1 [file peerj-11-16342-s001.pdf]

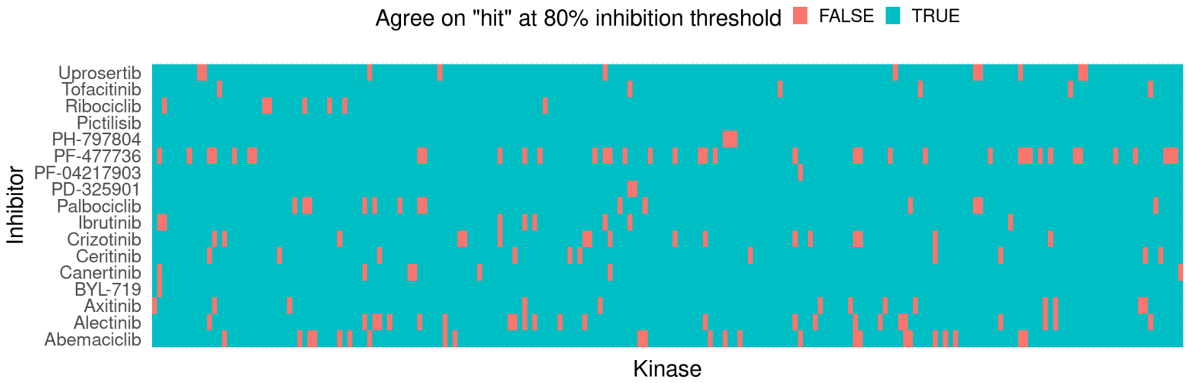

Supplementary Fig 1. Hit-calling accuracy comparison of kinobeads and KINOMEscan assays. Heatmap plot showing all different kinases (x-axis) that are targeted by a set of kinase inhibitors (y axis). Each cell on the heatmap represents the inhibition of that particular kinase by that particular drug. All the inhibitions were binarised at the 0.8 threshold, implying a “hit” if a kinase is inhibited more than 80% by a given drug. The colouring of each cell indicates if both the kinobeads and KINOMEscan assays agree that a given inhibitor-kinase pair is a “hit” or not. Cases where the two assays agree are coloured blue, while the orange cells denote where the two assays do not agree.
